# Treatment with β-blocker nebivolol ameliorates oxidative stress and endothelial dysfunction in tenofovir-induced nephrotoxicity in rats

**DOI:** 10.3389/fmed.2022.953749

**Published:** 2022-08-04

**Authors:** Mariana Moura Nascimento, Desiree Rita Denelle Bernardo, Ana Carolina de Bragança, Maria Heloisa Massola Shimizu, Antonio Carlos Seguro, Rildo Aparecido Volpini, Daniele Canale

**Affiliations:** ^1^Laboratorio de Investigacao Medica 12 (LIM12), Faculdade de Medicina, Universidade de São Paulo, São Paulo, Brazil; ^2^Laboratorio de Investigacao Medica 12 (LIM12), Faculdade de Medicina, Hospital das Clinicas HCFMUSP, Universidade de São Paulo, São Paulo, Brazil

**Keywords:** tenofovir, nephrotoxicity, nebivolol, hypertension, endothelium dysfunction, oxidative stress

## Abstract

**Background:**

Tenofovir disoproxil fumarate (TDF), a widely prescribed component in antiretroviral regimens, has been associated with nephrotoxicity. Nebivolol is a third generation selective β-1 adrenergic receptor blocker and may protect renal structure and function through the suppression of oxidative stress and enhancement of nitric oxide (NO) synthesis. We aimed to investigate whether nebivolol could be an effective therapeutic strategy to mitigate tenofovir-induced nephrotoxicity.

**Methods:**

We allocated Wistar rats to four groups: control (C), received a standard diet for 30 days; NBV, received a standard diet for 30 days added with nebivolol (100 mg/kg food) in the last 15 days; TDF, received a standard diet added with tenofovir (300 mg/kg food) for 30 days; and TDF+NBV, received a standard diet added with tenofovir for 30 days and nebivolol in the last 15 days.

**Results:**

Long-term exposure to tenofovir led to impaired renal function, induced hypertension, endothelial dysfunction and oxidative stress. Nebivolol treatment partially recovered glomerular filtration rate, improved renal injury, normalized blood pressure and attenuated renal vasoconstriction. Administration of nebivolol contributed to reductions in asymmetric dimethylarginine (ADMA) levels as well as increases in endothelial nitric oxide sintase (eNOS) accompanied by renin-angiotensin-aldosterone system downregulation and decreases in macrophage and T-cells infiltrate. Furthermore, nebivolol was responsible for the maintenance of the adequate balance of thiobarbituric acid reactive substances (TBARS) and glutathione (GSH) levels and it was associated with reductions in NADPH oxidase (NOX) subunits.

**Conclusion:**

Nebivolol holds multifaceted actions that promote an advantageous option to slow the progression of kidney injury in tenofovir-induced nephrotoxicity.

## Introduction

Human immunodeficiency virus (HIV) continues to be a major global public health issue, having claimed millions of lives so far (1). The large-scale use of highly active antiretroviral therapy (HAART) improved the life expectancy of individuals infected with HIV and lowered the incidence of AIDS-related diseases (2). Tenofovir disoproxil fumarate (TDF) is a nucleotide reverse transcriptase inhibitor considered a first-line antiretroviral drug and it is usually prescribed for the treatment of HIV and hepatitis B (HBV) based on its efficacy and tolerability in clinical trials (3–5). However, long-term exposure to tenofovir has been linked to renal failure, hypertension, abnormal lipid metabolism and oxidative stress (3). Tenofovir-induced nephrotoxicity was reported in approximately 15% of patients treated with this drug for 2–9 years (6). The adverse effects of tenofovir are mainly attributed to its involvement with the renin-angiotensin-aldosterone system (RAAS), nitric oxide (NO) signaling cascade and redox state balance (3, 7).

Nebivolol is a third generation selective β-1 adrenergic receptor blocker that also displays NO-mediated vasodilatory actions through β-3 receptor agonism (8). Due to this singular pharmacological profile, nebivolol exhibits several benefits that set it apart from other traditional β-blockers such as the improvement of renovascular parameters, metabolic syndrome, hemodynamics and oxidative stress (9). Nebivolol is currently used in the treatment of hypertension and cardiovascular diseases in the US and Europe, respectively (8). Previous experimental studies have shown that nebivolol protected against warm renal ischemia/reperfusion (10) and diabetic nephropathy (11). In addition, nebivolol presented beneficial effects in nephrotoxicity models of contrast media (12), gentamicin (13) and cyclosporine A (14). The mechanisms by which nebivolol may protect renal structure and function appears to be directly related to the reduction of oxidative stress and improvement of endothelial dysfunction, as well as its anti-inflammatory, anti-fibrotic and anti-apoptotic properties (10–14).

Considering that nebivolol is effective in a wide range of kidney illnesses, the aim of this study was to investigate whether the therapeutic actions of nebivolol could also be advantageous to mitigate tenofovir-induced nephrotoxicity.

## Materials and methods

### Experimental protocol

Male Wistar rats weighing 180–200 g were obtained from the animal facilities of the University of São Paulo—Institute of Biomedical Sciences. During the 30-day protocol, we kept our animals at controlled temperature (23 ± 1°C) with a light/dark cycle of 12/12 h. Rats received a standard diet (Nuvilab, PR, Brazil) added with tenofovir (300 mg/kg food equivalent to approximately 30 mg/kg BW) and/or nebivolol (100 mg/kg food equivalent to approximately 10 mg/kg BW) and had free access to tap water. Rats were allocated to the following groups: control, received a standard diet for 30 days; NBV, received a standard diet for 30 days added with nebivolol in the last 15 days; TDF, received a standard diet added with tenofovir for 30 days; and TDF+NBV, received a standard diet added with tenofovir for 30 days and nebivolol in the last 15 days. The chosen doses of tenofovir and nebivolol were based on previous experimental studies (7, 13, 15). We conducted all the experimental procedures in accordance with the guidelines outlined and approved by the local Research Ethics Committee (CEUA-HCFMUSP, protocol number 1287/2019).

### Analysis of urine samples

Before the clearance studies, all the rats were placed in individual metabolic cages. We collected 24-h urine to assess urinary output and then centrifuged the samples to remove suspended material. We evaluated urinary protein excretion (U_Prot_V, mg/day) by colorimetric system using a commercial kit (Labtest Diagnóstica, MG, Brazil).

### Inulin clearance and hemodynamic studies

On day 30, we anesthetized the animals with sodium thiopental (50 mg/kg BW) and then we cannulated the trachea with a PE-240 catheter for spontaneous breathing. The jugular vein was cannulated with PE-60 catheter for infusion of inulin and fluids. To monitor mean arterial pressure (MAP, mmHg) and collect blood samples, the right femoral artery was catheterized with a PE-50 catheter. We assessed MAP with a data acquisition system (MP100; Biopac Systems, CA, USA). To collect urine samples, we cannulated the bladder with a PE-240 catheter by suprapubic incision. After the surgical procedure, a loading dose of inulin (100 mg/kg BW diluted in 1 mL of 0.9% saline) was administered through the jugular vein. A constant infusion of inulin (10 mg/kg BW) was started and continued at 0.04 mL/min throughout the whole experiment. We collected three urine samples at 30-min intervals. Blood samples were obtained at the beginning and at the end of the experiment. Inulin clearance values represent the mean of three periods. Plasma and urinary inulin were determined by the anthrone method, and the glomerular filtration rate (GFR) data were expressed as mL/min/100g BW. To measure renal blood flow (RBF, mL/min), we made a median incision and dissected the left renal pedicle for isolating the renal artery. An ultrasonic flow probe was placed around the exposed renal artery, and RBF was measured with an ultrasonic flow meter (T402; Transonic Systems, MD, USA). We divided blood pressure by RBF to calculate renal vascular resistance (RVR, mmHg/mL/min).

### Biochemical parameters

We determined plasma levels of phosphate (${\text{PO}}_{4}^{3-}$, mg/dL), total cholesterol (cholesterol, mg/dL) and triglycerides (mg/dL) by colorimetric assay (Labtest Diagnóstica, MG, Brazil). We assessed plasma aldosterone (Aldo, pg/mL), angiotensin II (AngII, pg/mL) and asymmetric dimethylarginine (ADMA, ng/mL) by enzyme linked immunosorbent assay (ELISA) using commercial kits: Aldosterone (Enzo Life Sciences, NY, USA), Rat AngII and ADMA (Elabscience^®^, TX, USA). The detection system and the quantification followed the protocols described by the manufacturers.

### Tissue sample preparation

After blood samples collection, we perfused the kidneys with a phosphate-buffered solution (PBS, pH 7.4). We froze the right kidneys in liquid nitrogen and stored at −80°C for western blotting and ELISA. The left kidneys were removed and a fragment of the renal tissue was fixed in methacarn solution (60% methanol, 30% chloroform and 10% glacial acetic acid) for 24 h and replaced by 70% alcohol thereafter. The kidney blocks were embedded in paraffin and cut into 4-μm sections for histological and immunohistochemical (IHC) studies.

### Total protein isolation

Kidney samples were homogenized in ice-cold isolation solution (200 mM mannitol, 80 nM HEPES and 41 mM KOH, pH 7.5) containing a protease inhibitor cocktail (Sigma Chemical Company, MO, USA) with a homogenizer (Tissue Master TM125, Omni International, GA, USA). Homogenates were centrifuged at 4,000 × rpm for 30 min at 4°C to remove nuclei and cell debris. Supernatants were isolated and protein was quantified by Bradford assay (Bio-Rad Laboratories, CA, USA).

### Western blot assay

For Western blot analysis, 100 μg of total kidney protein were separated on SDS-polyacrylamide minigels by electrophoresis (16). After transfer by electroelution to PVDF membranes (GE Healthcare Limited, Little Chalfont, UK), blots were blocked for 1 h with 5% non-fat milk in Tris-buffered saline solution. Blots were then incubated with primary antibodies for anti-eNOS (BD Bioscience, CA, USA); anti-iNOS (MyBiosource, CA, USA); anti-HO-1 (AssayDesigns, MI, USA); anti-MnSOD and anti-Nrf2 (Cayman Chemicals, MI, USA); anti-p47^phox^ and anti-p67^phox^ (Santa Cruz Biotechnology, CA, USA). The labeling was visualized with a horseradish peroxidase-conjugated secondary antibody (anti-mouse or anti-rabbit, Sigma Chemical, MO, USA) and enhanced chemiluminescence detection (GE Healthcare Limited, Little Chalfont, UK). Kidney protein levels were further analyzed with a gel documentation system (Alliance 4.2; Uvitec, Cambridge, UK) and the software Image J for Windows (Image J-NIH Image). We used densitometry to quantitatively analyze the protein levels, normalizing the bands to β-actin expression (anti-β-actin, Sigma Chemical, MO, USA).

### ELISA in renal tissue

We assessed AngII (pg/μg protein), Monocyte Chemotactic Protein 1 (MCP-1/CCL2, ng/μg protein), Transforming Growth Factor Beta 1 (TGF-β1, ng/μg protein) and Collagen Type 3 (COL3, pg/μg protein) in renal tissue by ELISA using commercial kits (Elabscience^®^, TX, USA). The detection system and quantification followed the protocols described by the manufacturer. The absorbances were obtained using the Epoch/2 device (Biotek Instruments, VE, USA).

### Light microscopy and IHC analysis

Four-micrometer histological sections of kidney tissue were stained with Hematoxylin-eosin (HE) and examined under light microscopy. For the evaluation of renal damage, 40–60 grid fields (× 400 magnification) measuring 0.245 mm^2^ were evaluated by graded scores according to the following criteria: (0), <5% of the field showing tubular epithelial swelling, vacuolar degeneration, necrosis, and desquamation; (I), 5–25% of the field presenting renal lesions; (II), involvement of 25–50% with renal damage; (III), 50–75% of damaged area; and (IV), more than 75% of the grid field presenting renal lesions. To minimize bias during the morphometric examination, the observer was blinded to the treatment groups. The mean score for each rat and the mean score for each group were calculated (17, 18). For histomorphometry, the images obtained by microscopy were captured on a computer screen via an image analyzer software (ZEN, Carl Zeiss, Munich, Germany). Immunohistochemistry was performed on 4-μm-thick paraffinized kidney sections mounted on 2% silane-coated glass slides. We used the following antibodies: anti-CD68 (AbD Serotec, Oxford, UK); anti-CD3 (Dako, Glostrup, Denmark); and anti-aminopeptidase P (JG12, Santa Cruz Biotechnology, CA, USA). We subjected the kidney tissue sections to IHC reaction according to the protocol for each primary antibody. Reaction products were detected by anti-rabbit or mouse EnVision+ System™ and the color reaction was developed in 3,3-diaminobenzidine (Dako North America, CA, USA). Counterstaining was with Harris' hematoxylin. We analyzed 30–40 renal cortex fields (0.09 mm^2^) to evaluate the immunoreactions. The volume ratios of positive areas of renal tissue (%), determined by the color limit, were obtained by ZEN image analyzer software (Carl Zeiss, Munich, Germany) on a computer coupled to a microscope (Carl Zeiss Axioskop 40) and a digital camera (19, 20). To minimize bias during the IHC analysis, the observer was blinded to the treatment groups.

### Reactive oxygen metabolites assessment

Plasma (nmol/mL), urinary (μmol/mg creatinine) and tissue (nmol/mg protein) levels of thiobarbituric acid reactive substances (TBARS) were assessed using TBARS Assay Kit (Cayman Chemicals, MI, USA). The detection system and quantification followed the protocols provided by the manufacturer. The absorbances were obtained using the Epoch/2 device (Biotek Instruments, VE, USA). Glutathione (GSH) was determined in total blood by the method of Sedlak and Lindsay (21). Whole blood was processed by addition of four volumes of ice-cold 5% (W/V) metaphosphoric acid (Sigma Chemical, St. Louis, MO, USA) and centrifuged at 14,000 × g for 10 min. This assay consists of reacting the supernatants of the total blood with Ellman's reagent to produce a yellow pigment measured spectrophotometrically at 412 nm. The GSH was quantified by mean of standard curve and reported as μmol/mL (22).

### Statistical analysis

All quantitative data were expressed as mean ± SEM. Differences among groups were analyzed with GraphPad Prism 5.0 software (GraphPad Software, CA, USA) by one-way analysis of variance followed by the Student–Newman–Keuls test. Values of *p* < 0.05 were considered statistically significant.

## Results

### Renal function, hemodynamic analysis and biochemical studies

Tenofovir-treated rats presented a significantly impaired renal function compared to C and NBV rats. TDF+NBV group showed a partial recovery of GFR compared to TDF group (Table 1). In addition to an impaired renal function, TDF group exhibited a higher MAP, a diminished RBF and an augmented RVR compared to C and NBV groups. Treatment with nebivolol lowered MAP, slightly increased RBF and decreased RVR in the TDF+NBV rats compared to the TDF rats (Table 1). Furthermore, TDF group exhibited a lower concentration of ${\text{PO}}_{4}^{3-}$ compared to C and NBV rats. Treatment with nebivolol reestablished phosphatemia in the TDF+NBV group compared to the C and NBV groups (Table 2). TDF group showed higher plasma levels of cholesterol and triglycerides compared to C group. These parameters were significantly reduced in the TDF+NBV rats compared to the TDF rats (Table 2).

**Table 1:** Renal function and hemodynamic parameters evaluated after the 30-day protocol.

	C (*n* = 9)	NBV (*n* = 9)	TDF (*n* = 10)	TDF+NBV (*n* = 10)
GFR (mL/min/100g)	0.91 ± 0.04	0.82 ± 0.04	0.40 ± 0.04^a^^d^	0.74 ± 0.03^b^^g^
MAP (mmHg)	116 ± 4	119 ± 5	146 ± 3^a^^d^	117 ± 2^g^
RBF (mL/min)	7.03 ± 0.10	6.78 ± 0.13	5.57 ± 0.06^a^^d^	5.89 ± 0.15^a^^d^^i^
RVR (mmHg/mL/min)	16.53 ± 0.63	17.83 ± 0.79	26.05 ± 0.46^a^^d^	20.71 ± 0.87^a^^f^^g^

*C, control; NBV, Nebivolol; TDF, Tenofovir; GRF, glomerular filtration rate; MAP, mean arterial pressure; RBF, renal blood flow; and RVR, renal vascular resistance*.

*Values are means ± SEM*.

a *p < 0.001;*

b *p < 0.01 vs. C;*

d *p < 0.001;*

f *p < 0.05 vs. NBV;*

g *p < 0.001 and*

i *p < 0.05 vs. TDF*.

**Table 2:** Biochemical measurements and inflammatory/fibrosis markers evaluated after the 30-day protocol.

	C (*n* = 9)	NBV (*n* = 9)	TDF (*n* = 10)	TDF+NBV (*n* = 10)
Plasma				
${\text{PO}}_{4}^{3-}$ (mg/dL)	6.72 ± 0.21	7.09 ± 0.24	5.04 ± 0.14^a^^d^	6.12 ± 0.40^h^
Cholesterol (mg/dL)	39.05 ± 2.76	46.22 ± 2.48	51.72 ± 2.19^c^	39.16 ± 3.73^i^
Triglycerides (mg/dL)	46.07 ± 9.91	73.98 ± 14.42	158.40 ± 19.33^a^^e^	111.20 ± 11.14^b^^i^
AngII (pg/mL)	138.8 ± 12.0	110.1 ± 6.9	186.8 ± 14.7^c^^e^	147.8 ± 11.0^i^
Aldo (pg/mL)	1,128 ± 141	1,532 ± 98	2,970 ± 192^a^^d^	1,903 ± 190^b^^g^
ADMA (ng/mL)	57 ± 3	58 ± 6	74 ± 4^c^^f^	55 ± 4^i^
TBARS (nmol/mL)	2.46 ± 0.21	2.92 ± 0.20	3.97 ± 0.42^a^^f^	2.47 ± 0.15^g^
GSH (μmol/mL)	2.84 ± 0.36	2.41 ± 0.44	1.00 ± 0.26^c^^f^	2.19 ± 0.37^i^
Urine				
U_Prot_V (mg/day)	9.3 ± 1.4	11.9 ± 0.8	18.4 ± 1.1^a^^d^	14.8 ± 0.4^a^^f^^h^
TBARS (μmol/mg creatinine)	0.184 ± 0.004	0.210 ± 0.009	0.253 ± 0.010^a^^f^	0.173 ± 0.011^g^
Renal tissue				
AngII (pg/μg protein)	3.84 ± 1.19	4.51 ± 1.01	8.29 ± 1.31^c^^f^	3.82 ± 0.74^i^
TBARS (nmol/μg protein)	13.22 ± 1.07	15.20 ± 1.73	19.63 ± 1.24^c^^f^	14.38 ± 0.94^i^
MCP-1 (ng/μg protein)	0.36 ± 0.03	0.38 ± 0.03	0.52 ± 0.04^c^^f^	0.43 ± 0.03^i^
TGF-β1 (ng/μg protein)	0.10 ± 0.02	0.11 ± 0.01	0.22 ± 0.02^b^^e^	0.10 ± 0.02^h^
COL3 (pg/μg protein)	63.9 ± 9.1	67.9 ± 5.9	93.3 ± 9.7^c^^f^	63.9 ± 2.7^i^

*C, control; NBV, Nebivolol; TDF, Tenofovir. ${\text{PO}}_{4}^{3-}$, phosphate; Cholesterol, total cholesterol; AngII, angiontensin II; Aldo, aldosterone; ADMA, asymmetric dimethylarginine; TBARS, thiobarbituric acid reactive substances; GSH, glutathione; U_Prot_V, urinary protein excretion; MCP-1, monocyte chemotactic protein 1; TGF-β1, transforming growth factor β1; COL3, collagen type III*.

*Values are means ± SEM*.

a *p < 0.001;*

b *p < 0.01;*

c *p < 0.05 vs. C;*

d *p < 0.001;*

e *p < 0.01;*

f *p < 0.05 vs. NBV;*

g *p < 0.001;*

h *p < 0.01, and*

i *p < 0.05 vs. TDF*.

### Effects of nebivolol on hypertension, RAAS and NO signaling pathway in TDF-induced nephrotoxicity

**Figure 1:**
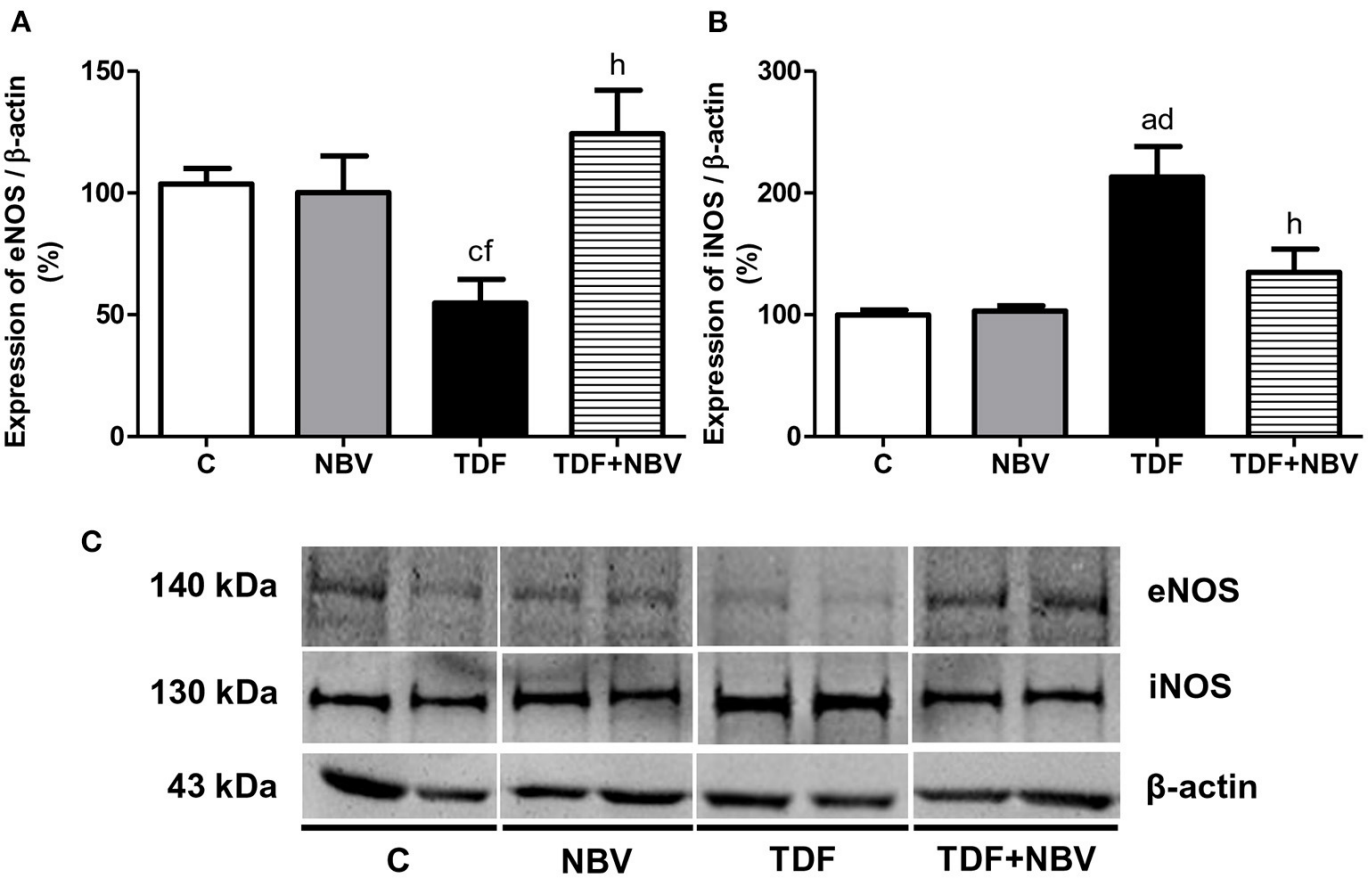
Semiquantitative immunoblotting for eNOS and iNOS expression in rat kidney tissue. **(A)** eNOS densitometric analysis of samples from C (*n* = 7), NBV (*n* = 7), TDF (*n* = 8) and TDF+NBV (*n* = 8) rats. **(B)** iNOS densitometric analysis of samples from C (*n* = 7), NBV (*n* = 7), TDF (*n* = 8) and TDF+NBV (*n* = 8) rats. **(C)** Representative immunoblots which reacted with anti-eNOS and anti-iNOS revealing bands of 140 and 130kDa, respectively. Values are means ± SEM. ^a^*p* < 0.001, ^c^*p* < 0.05 vs. C; ^d^*p* < 0.001, ^f^*p* < 0.05 vs. NBV; ^h^*p* < 0.01 vs. TDF. C, control; NBV, Nebivolol; TDF, Tenofovir.

**Figure 2:**
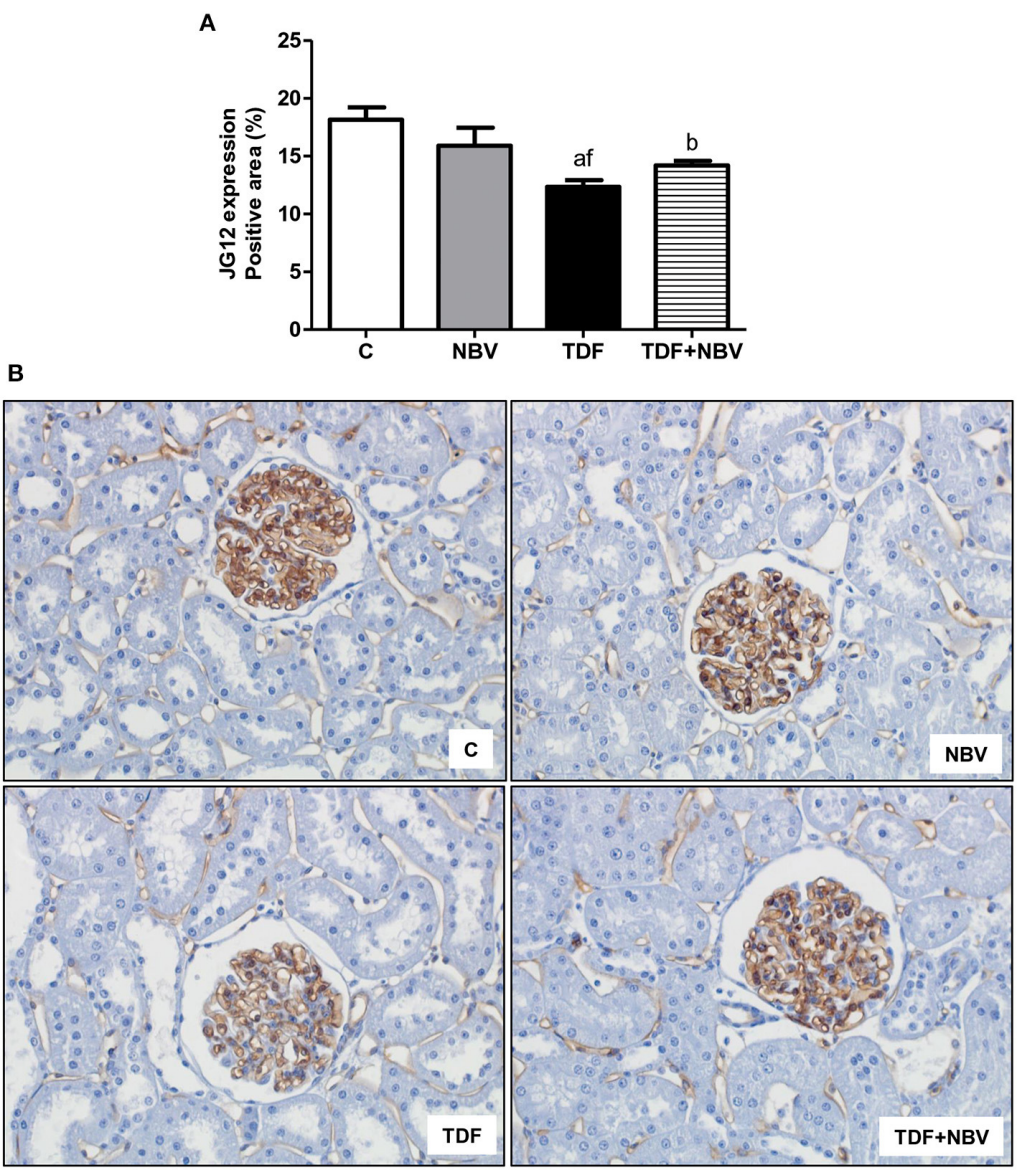
Immunohistochemical analysis for aminopeptidase P (JG12) expression by glomerular tuft area in rat kidney tissue. **(A)** Bar graph of JG12 expression values. **(B)** Representative photomicrographs of immunostaining for JG12 from a C (*n* = 7), NBV (*n* = 7), TDF (*n* = 8) and TDF+NBV (*n* = 8) rat (× 400). Values are means ± SEM. ^a^*p* < 0.001, ^b^*p* < 0.01 vs. C; ^f^*p* < 0.05 vs. NBV. C, control; NBV, Nebivolol; TDF, Tenofovir.

Hypertension was accompanied by alterations in the RAAS. Tenofovir-treated rats presented increased plasma level and renal tissue expression of AngII as well as plasma Aldo concentration compared to C and NBV rats. Administration of nebivolol restored both components of the RAAS in the TDF+NBV rats compared to the C and NBV rats (Table 2). Following renovascular studies, we observed higher ADMA plasma levels in the TDF rats compared to the C and NBV rats. TDF+NBV group showed an improvement in ADMA plasma concentration compared to TDF group (Table 2). Corroborating this data, tenofovir-treated rats exhibited a downregulated and an upregulated renal protein expression of eNOS and iNOS, respectively, compared to C and NBV rats. TDF+NBV group presented a restoration of eNOS and iNOS renal expression compared to C and NBV groups (Figure 1). In addition, JG12 staining per glomerular tuft area was significantly decreased in the TDF rats compared to the C and NBV rats. TDF+NBV group exhibited a slightly tendency for upregulation of JG12 expression compared to TDF group (Figure 2).

### Treatment with nebivolol ameliorated kidney damage, renal fibrosis formation and inflammation

**Figure 3:**
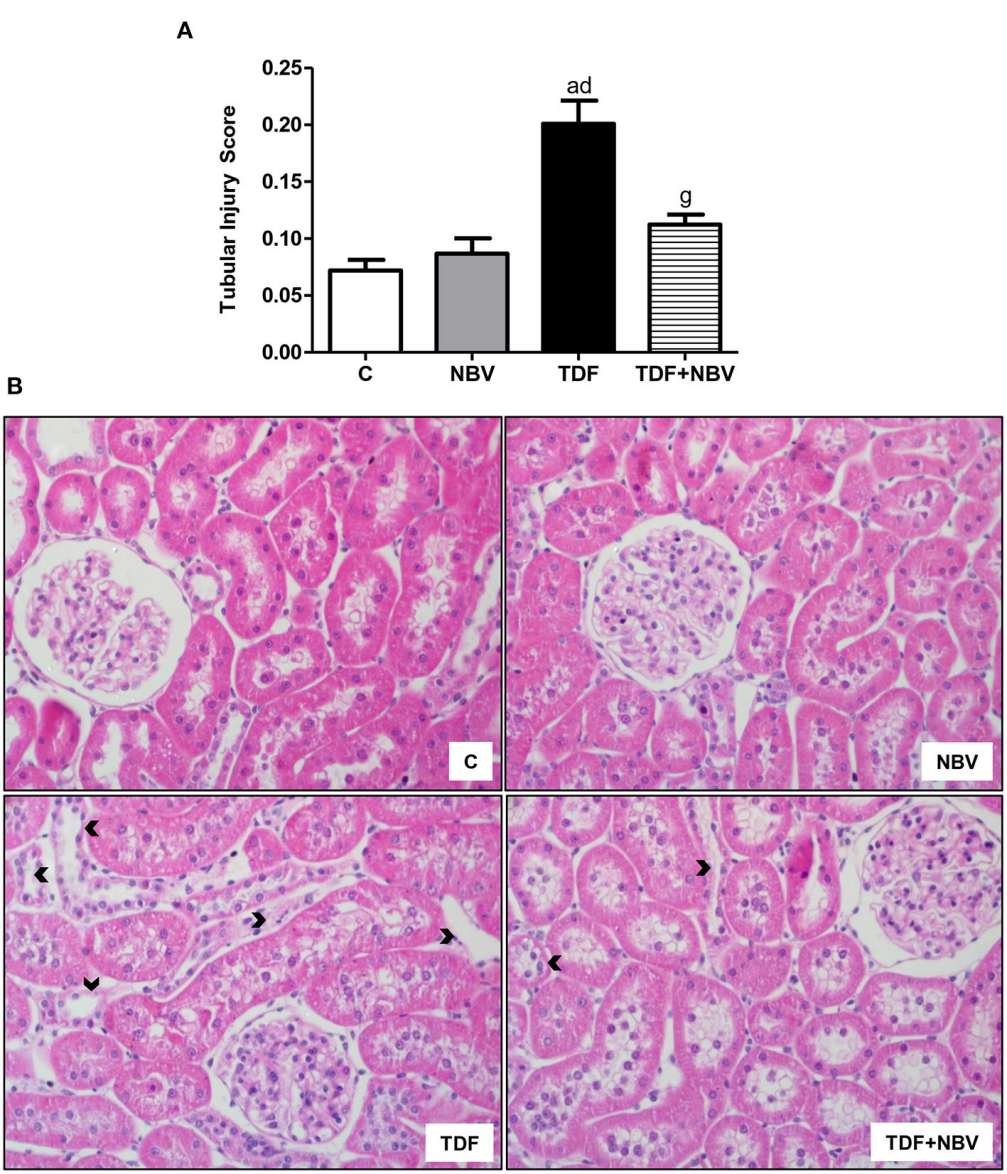
Tubular injury score in the renal cortex evaluated at the end of the protocol. **(A)** Bar graph of tubular injury score values. **(B)** Representative photomicrographs of renal histological changes from a C (*n* = 7), NBV (*n* = 7), TDF (*n* = 8) and TDF+NBV (*n* = 8) rat (× 400). Arrowheads: morphological alterations including tubular cell necrosis, areas of denuded basement membrane, flattening of proximal tubular cells with brush border loss and tubular atrophy or dilatation. Values are means ± SEM. ^a^*p* < 0.001 vs. C; ^d^*p* < 0.001 vs. NBV; ^g^*p* < 0.001 vs. TDF. C, control; NBV, Nebivolol; TDF, Tenofovir.

**Figure 4:**
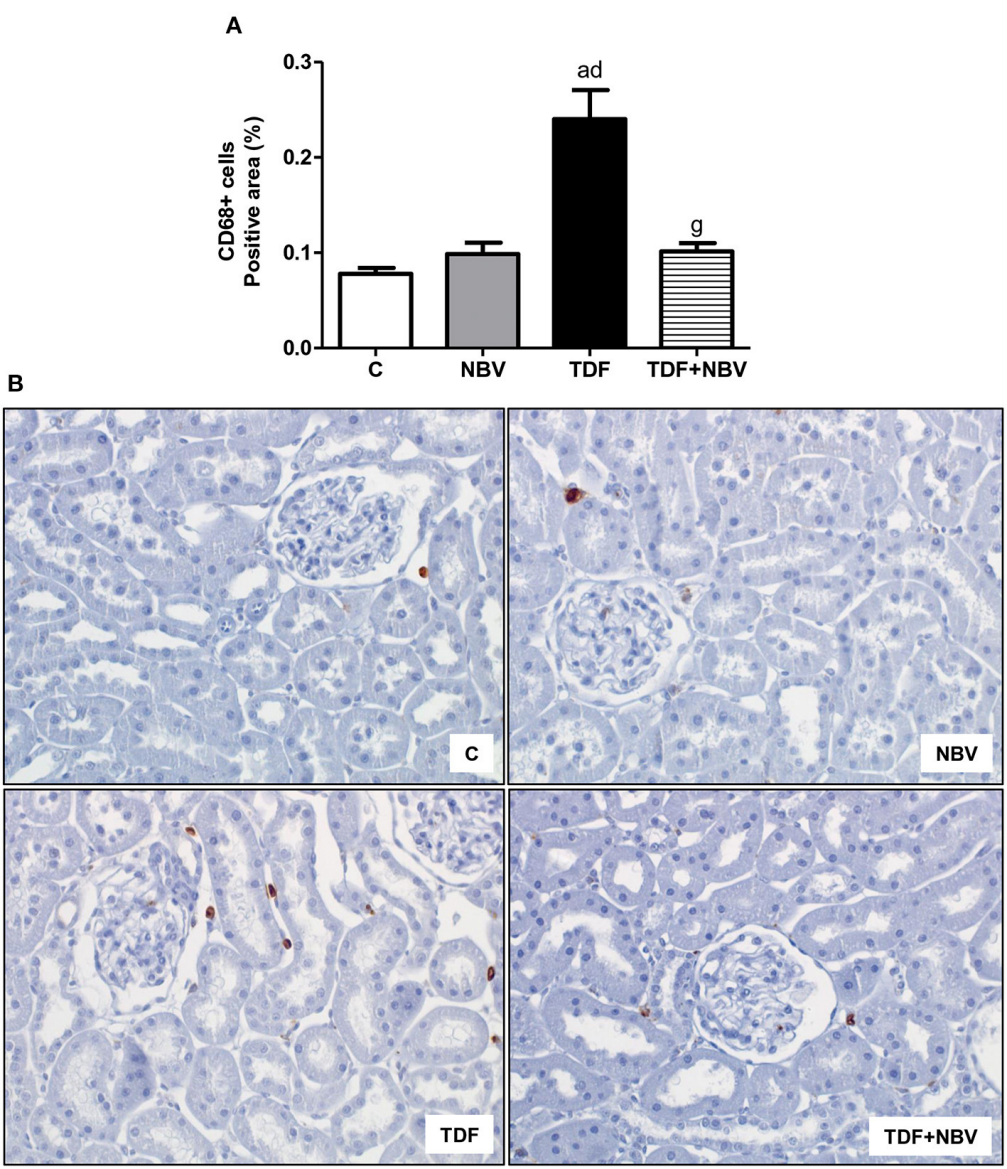
Immunohistochemical analysis for CD68+ cells expression in rat kidney tissue. **(A)** Bar graph of CD68 positive cells expression values. **(B)** Representative photomicrographs of immunostaining for CD68 positive cells in the renal cortex from a C (*n* = 7), NBV (*n* = 7), TDF (*n* = 8) and TDF+NBV (*n* = 8) rat (× 400). Values are means ± SEM. ^a^*p* < 0.001 vs. C; ^d^*p* < 0.001 vs. NBV; ^g^*p* < 0.001 vs. TDF. C, control; NBV, Nebivolol; TDF, Tenofovir.

**Figure 5:**
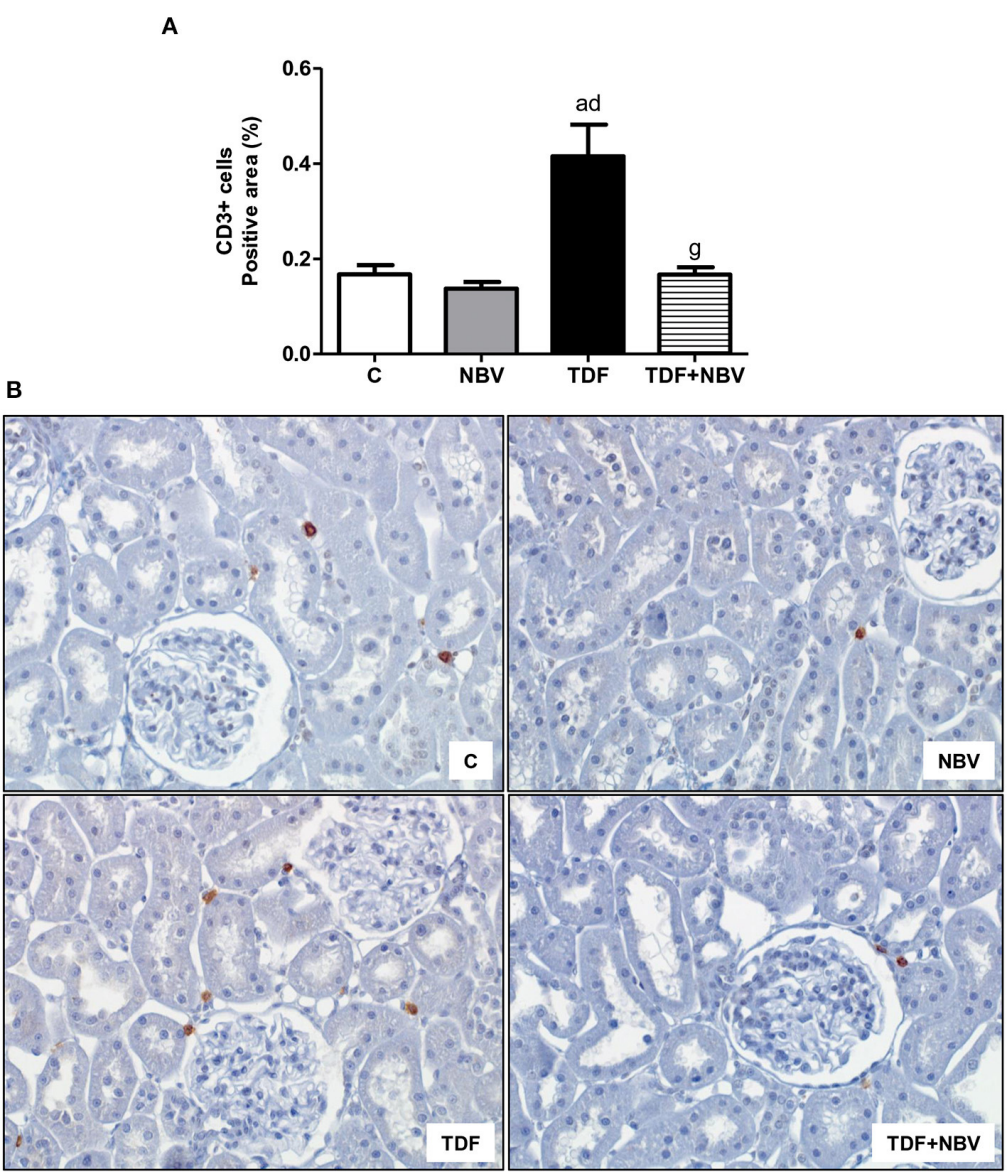
Immunohistochemical analysis for CD3+ cells expression in rat kidney tissue. **(A)** Bar graph of CD3 positive cells expression values. **(B)** Representative photomicrographs of immunostaining for CD3 positive cells in the renal cortex from a C (*n* = 7), NBV (*n* = 7), TDF (*n* = 8) and TDF+NBV (*n* = 8) rat (× 400). Values are means ± SEM. ^a^*p* < 0.001 vs. C; ^d^*p* < 0.001 vs. NBV; ^g^*p* < 0.001 vs. TDF. C, control; NBV, Nebivolol; TDF, Tenofovir.

TDF rats showed mild morphological alterations including tubular cell necrosis, areas of denuded basement membrane, flattening of proximal tubular cells with brush border loss and tubular atrophy or dilatation. TDF+NBV group exhibited a significantly decrease in tubular injury score compared to TDF group (Figure 3). Moreover, tenofovir-treated rats showed a greater loss of urinary protein compared to C and NBV rats. Administration of nebivolol reduced U_prot_V in the TDF+NBV rats compared to the TDF rats (Table 2). TDF group showed a higher renal concentration of TGF-β1 compared to C and NBV groups. Treatment with nebivolol lowered this parameter in the TDF+NBV group compared to the TDF group since tubular injury score was also restored (Table 2). TDF rats presented an increase in renal expression of COL3 compared to C and NBV rats. Administration of nebivolol led to a reduction in COL3 renal expression in the TDF+NBV group compared to the TDF rats (Table 2). Tenofovir-treated rats showed a higher amount of MCP-1 compared to C and NBV rats. TDF+NBV group exhibited a remarkable reduction in this parameter compared to TDF rats (Table 2). In accordance with our data regarding MCP-1, we observed a higher renal expression of CD68+ and CD3+ cells in the TDF rats compared to the C and NBV rats. TDF+NBV group presented a significant reduction in both renal expressions of CD68+ and CD3+ cells compared to TDF group (Figures 4, 5).

### Modulatory effect of nebivolol on oxidative stress: Role of NOX (NADPH oxidase) and Nrf2/HO-1 signaling pathways

**Figure 6:**
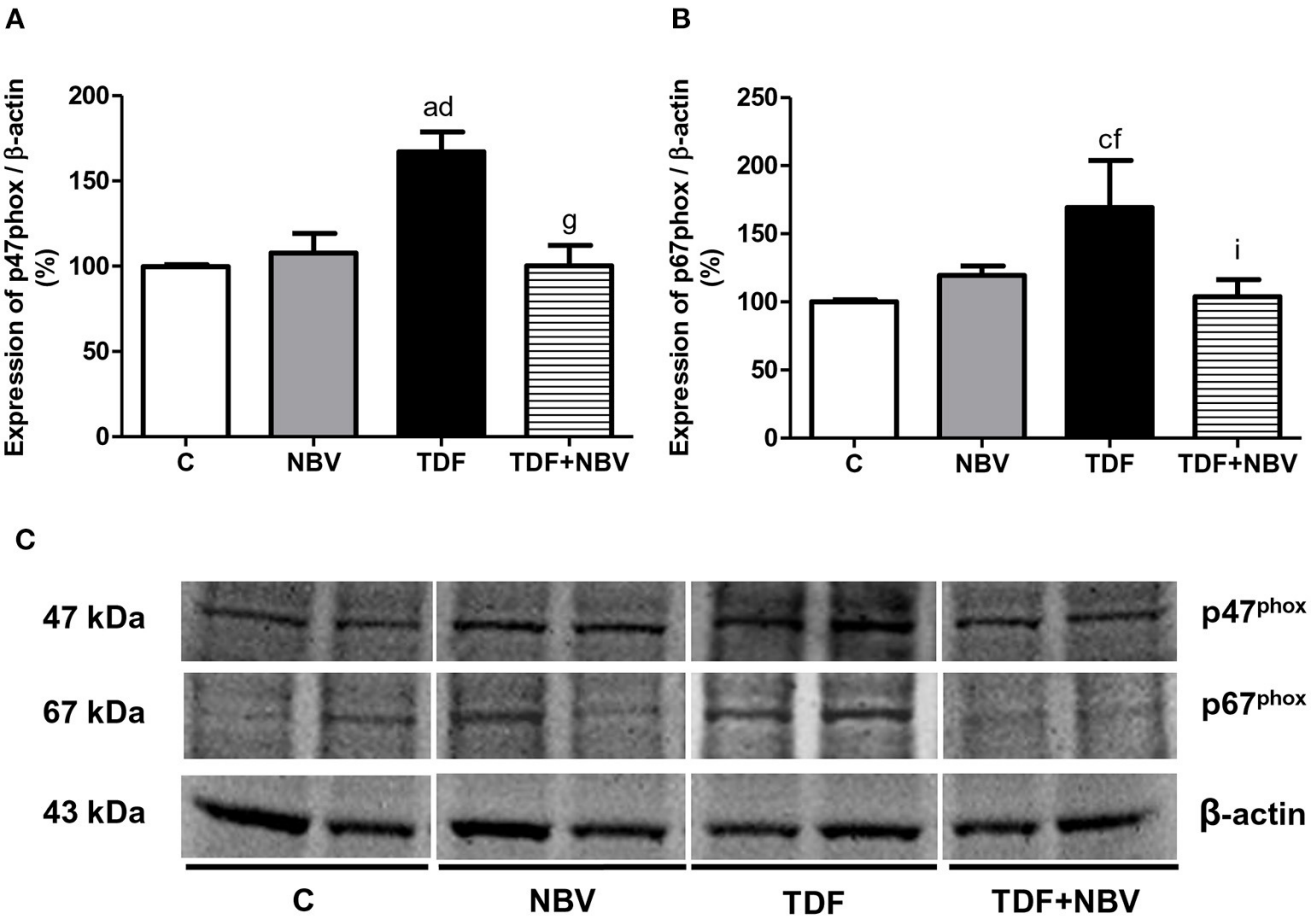
Semiquantitative immunoblotting for p47^phox^ and p67^phox^ expression in rat kidney tissue. **(A)** p47^phox^ densitometric analysis of samples from C (*n* = 7), NBV (*n* = 7), TDF (*n* = 8) and TDF+NBV (*n* = 8) rats. **(B)** p67^phox^ densitometric analysis of samples from C (*n* = 7), NBV (*n* = 7), TDF (*n* = 8) and TDF+NBV (*n* = 8) rats. **(C)** Representative immunoblots which reacted with anti-p47^phox^ and anti-p67^phox^ revealing bands of 47 and 67kDa, respectively. Values are means ± SEM. ^a^*p* < 0.001, ^c^*p* < 0.05 vs. C; ^d^*p* < 0.001, ^f^*p* < 0.05 vs. NBV; ^g^*p* < 0.001, ^i^*p* < 0.05 vs. TDF. C, control; NBV, Nebivolol; TDF, Tenofovir.

**Figure 7:**
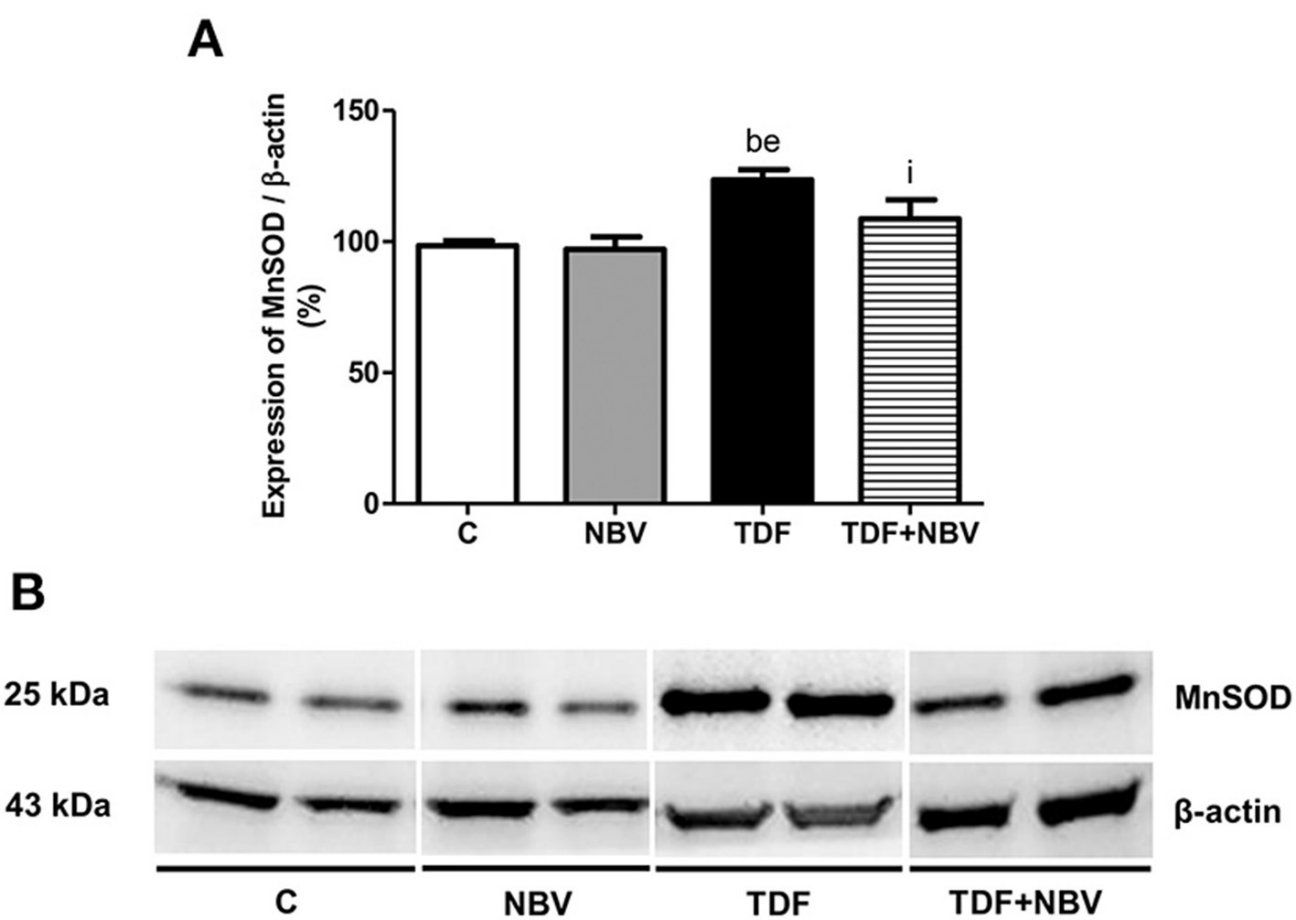
Semiquantitative immunoblotting for MnSOD expression in rat kidney tissue. **(A)** MnSOD densitometric analysis of samples from C (*n* = 7), NBV (*n* = 7), TDF (*n* = 8) and TDF+NBV (*n* = 8) rats. **(B)** Representative immunoblots which reacted with anti-MnSOD revealing a band of 25kDa. Values are means ± SEM. ^b^*p* < 0.01 vs. C; ^e^*p* < 0.01 vs. NBV; ^i^*p* < 0.05 vs. TDF. C, control; NBV, Nebivolol; TDF, Tenofovir.

**Figure 8:**
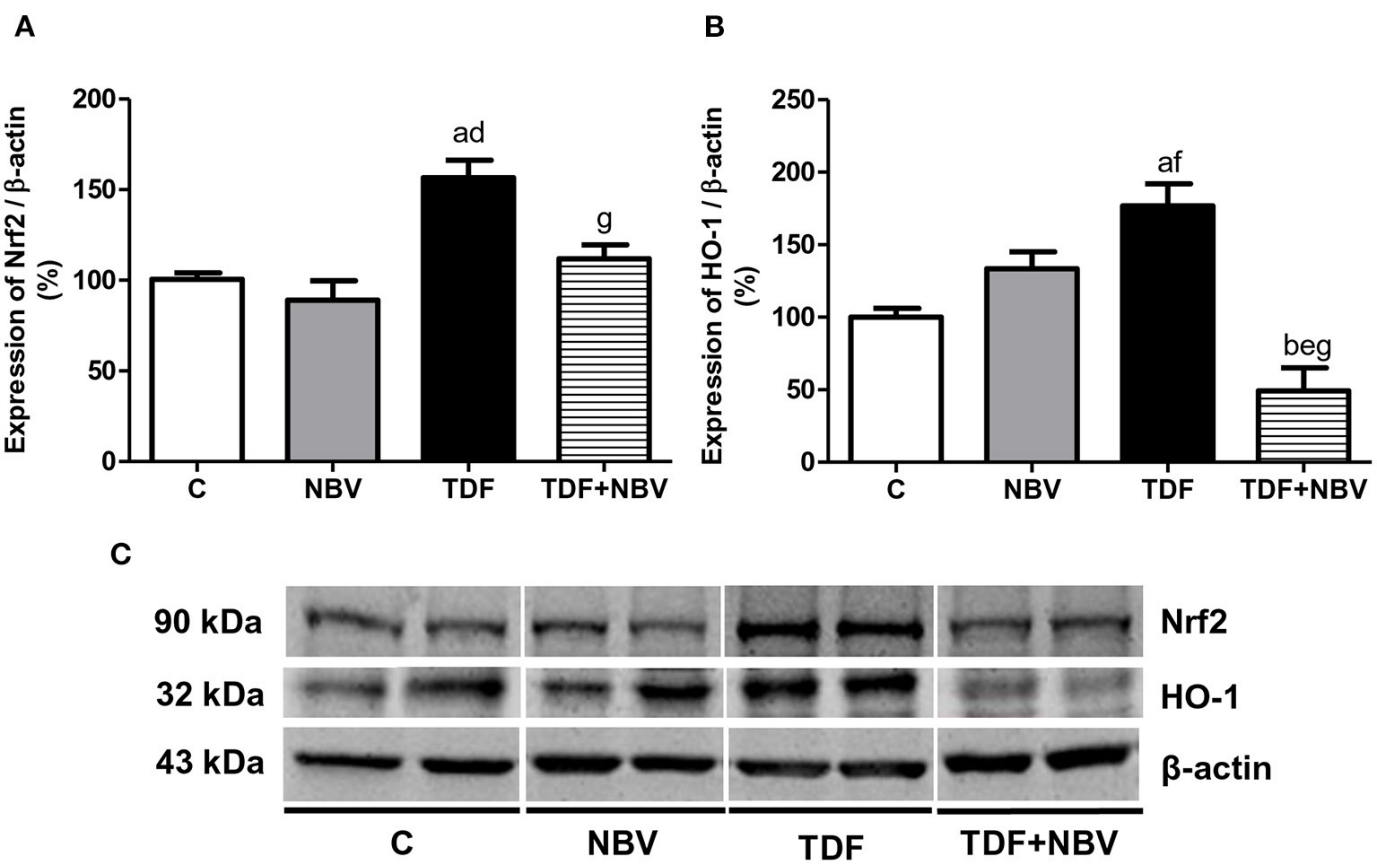
Semiquantitative immunoblotting for Nrf2 and HO-1 expression in rat kidney tissue. **(A)** Nrf2 densitometric analysis of samples from C (*n* = 7), NBV (*n* = 7), TDF (*n* = 8) and TDF+NBV (*n* = 8) rats. **(B)** HO-1 densitometric analysis of samples from C (*n* = 7), NBV (*n* = 7), TDF (*n* = 8) and TDF+NBV (*n* = 8) rats. **(C)** Representative immunoblots which reacted with anti-Nrf2 and anti-HO-1 revealing bands of 90 and 32kDa, respectively. Values are means ± SEM. ^a^*p* < 0.001, ^b^*p* < 0.01 vs. C; ^d^*p* < 0.001, ^e^*p* < 0.01, ^f^*p* < 0.05 vs. NBV; ^g^*p* < 0.001 vs. TDF. C, control; NBV, Nebivolol; TDF, Tenofovir.

Tenofovir-treated rats exhibited higher plasma, urinary and renal tissue TBARS levels compared to C and NBV rats. TDF+NBV group showed a restoration of all the TBARS parameters compared to C and NBV groups (Table 2). TDF rats showed a diminished plasma GSH concentration compared to C and NBV rats. TDF+NBV group presented a remarkable increase in plasma GSH levels compared to TDF group (Table 2). In addition, we found higher renal protein expressions of p47^phox^ and p67^phox^ in the TDF group compared to the C and NBV groups. Treatment with nebivolol notably reduced the renal expression of those enzymes necessary for NOX activity in the TDF+NBV rats compared to the TDF rats (Figure 6). Tenofovir-treated rats exhibited a higher MnSOD renal protein abundance compared to C and NBV groups. Strikingly, treatment with nebivolol reduced the renal expression of MnSOD in the TDF+NBV group compared to the TDF group (Figure 7). Similarly, we observed higher renal protein expressions of Nrf2 and HO-1 in the TDF group compared to the C and NBV groups. TDF+NBV rats presented a significant decrease in the expression of both Nrf2 and HO-1 compared to the TDF rats (Figure 8).

## Discussion

The number of HIV-infected individuals has been increasing in recent years, affecting mainly underdeveloped countries (1). Long-term exposure to tenofovir is associated with nephrotoxicity, leading to acute kidney injury (AKI) or chronic kidney disease (CKD) (4). Supporting previous data (3, 7), our current study demonstrated that tenofovir-treated rats presented impaired renal function, hypophosphatemia, hypertension, dyslipidemia, endothelial dysfunction and oxidative stress. Our results revealed that nebivolol improved renal function, normalized MAP, increased RBF and decreased RVR in the TDF+NBV rats. In addition, administration of nebivolol ameliorated renal injury and inflammation, suppressed RAAS activity, and normalized the parameters involved in NO signaling pathway and redox state.

Nebivolol attenuated renal injury in several experimental models regardless of its classic antihypertensive action (11, 12, 14). Our data showed that treatment with nebivolol increased GFR and reduced proteinuria in the TDF+NBV rats. This action is probably related to the specific pharmacological effect of nebivolol on the ability to induce NO bioavailability, improve antioxidant defenses, and reduce renal fibrosis (11, 23). Moreover, Wang et al. reported that Zucker diabetic fatty (ZDF) rats treated with nebivolol exhibited a decrease in tubular damage and glomerular basement membrane thickening and a restoration of podocytes, resulting in increased creatinine clearance and reduced proteinuria (24).

Proteinuria usually occurs along with hypertension in CKD patients. Hypertension is a well-known risk factor related to cardiovascular impairment and kidney disease progression (25). Experimental (11, 24, 26) and clinical trials (27) demonstrated that administration of nebivolol lowered MAP and reversed endothelial dysfunction. Our findings are in agreement with these observations and underscore the importance of nebivolol therapy in the maintenance of MAP. TDF+NBV group presented a normalization of MAP, which was accompanied by a decrease in both plasma and tissue concentrations of AngII and plasma Aldo levels. AngII, one of the main components of the RAAS, is a potent systemic vasoconstrictor responsible for Aldo secretion and the arising of hypertension, promoting renal mass loss and worsening of CKD (28–30). Blumenfeld et al. stated that β-adrenergic receptor blockers are frequently neglected as potent suppressors of renin secretion and, consequently, AngII formation. Corroborating our data, those authors demonstrated that plasma renin activity and AngII/Aldo plasma levels were remarkably reduced in hypertensive patients under regular treatment with β-blockers (31).

In addition to MAP restoration, TDF+NBV rats showed a significant increase in RBF and an important decrease in RVR. It is acknowledged that reduced NO production or availability is related to endothelial dysfunction, hypertension and progression of CKD (32, 33). Renal NO is synthesized primarily by eNOS and nNOS. Endothelial nitric oxide synthase is responsible for maintaining GFR and RBF while nNOS preserves glomerular hemodynamics. However, NO produced by iNOS can lead to apoptosis and lipid peroxidation (26). Thus, it is plausible to assume that the regulation of NOS isoforms by nebivolol contributes to its protective effects on the kidney. Our study demonstrated that the improvement in renal hemodynamic was possibly associated with an upregulation of eNOS and a downregulation of iNOS, indicating that the treatment with nebivolol induced vasodilation through the modulation of these enzymes in endothelial cells. Another mechanism involved with endothelial dysfunction includes the increase in plasma ADMA, an endogenous competitive inhibitor of NOS isoforms. Higher plasma ADMA levels are also linked to oxidative stress (32, 34). Corroborating previous data (24, 26), our results showed that tenofovir-treated animals exhibited augmented plasma ADMA concentration which was reversed with the administration of nebivolol. These findings suggest that ADMA is an important marker of endothelial dysfunction and its lower levels were probably responsible for the normalization of eNOS and iNOS renal protein expression in the TDF+NBV group. Another specific marker for vascular endothelium damage is JG12. TDF rats showed a significant decrease in JG12 expression in the glomerular capillaries. TDF+NBV rats presented an upward tendency regarding JG12 expression in glomerular vascular endothelium, which possibly contributed to the improvement of renal function in these rats. The combination of the beneficial effects of nebivolol on the RAAS and NO cascade results in renal protection and justifies its efficiency in reducing MAP and improving hemodynamic parameters.

Vascular endothelial damage triggers several pathological alterations displayed in the kidney disease progression (35). Treatment with nebivolol was also able to reverse kidney injury caused by long-term use of tenofovir, at least in part, due to its actions on pro-fibrotic molecules and inflammatory markers. Toblli et al. reported that ZDF rats exhibited large expressions of TGF-β1, collagen types I and III, MCP-1 and CD68+ cells and these alterations were attenuated after administration of nebivolol (11). In support of these findings, our study demonstrated that TDF+NBV rats presented lower TGF-β1 and COL3 renal expression. Likewise, treatment with nebivolol reestablished MCP-1 amounts, CD68+ and CD3+ cells expression in the renal tissue of TDF+NBV rats compared to C and NBV rats. Of note, suppression of TGF-β1 may be due to an increase in NO, which corroborates data previously described regarding the involvement of nebivolol in the NO cascade (36). Therefore, our results show a protective role of NBV in the modulation of both ECM components and inflammatory cells expression in tenofovir-induced nephrotoxicity.

Metabolic syndrome is characterized by hypertension, impaired glucose tolerance and dyslipidemia, and increases the risk of cardiovascular diseases and CKD (11). Previous studies have shown that regular exposure to tenofovir leads to higher levels of cholesterol and triglycerides (3, 37, 38). Similarly, our results demonstrated that tenofovir-treated rats presented dyslipidemia and treatment with nebivolol promoted a favorable effect on the lipid profile. Corroborating our data, experimental and clinical trials revealed that new generation β-blockers with NO-mediating vasodilating properties efficiently diminished MAP and had similar beneficial impact on glucose and insulin levels as well as on triglycerides and cholesterol concentration (11, 39). A possible explanation for this action of nebivolol on lipid metabolism could be associated with oxidative stress modulation (40).

Oxidant elements play an important role in the pathogenesis of tenofovir-induced nephrotoxicity (3) with NOX emerging as the crucial cytosolic source of reactive oxygen species (ROS) generation (41). Reactive oxygen species are highly reactive molecules responsible for the renal damage induced by RAAS upregulation and the oxidation of lipids and proteins, leading to glomerular and tubular injury and the onset of proteinuria. Reactive oxygen species also promote the uncoupling of eNOS, suppressing the activity of this enzyme and causing a reduction in the bioavailability of NO (42). Moreover, an imbalance between ROS production and antioxidant defense systems causes oxidative stress (43). Our findings showed that tenofovir administration led to an increase in lipid peroxidation and a decrease in the main intracellular antioxidant, as evidenced by higher plasma/urinary/tissue TBARS concentration and lower plasma GSH levels. These results suggest the influence of oxidative stress in the development of kidney injury and the onset of hypertension. In agreement with our data aforementioned, TDF animals exhibited a significant increase in both p47^phox^ and p67^phox^ NOX subunits protein expression. It is well-known that NOX is the predominant source for renal oxidative stress (23) and p47^phox^ is the most important subunit for the modulation of its activity (44). Treatment with nebivolol restored the parameters related to oxidative stress in TDF+NBV rats compared to C and NBV rats. These findings suggest that nebivolol contributed to maintain redox state balance with subsequent improvements in renal function and hemodynamics in this experimental model. Corroborating our results, Whaley-Connell et al. reported that the administration of nebivolol normalized endothelial function, reduced the activity of NOX and its subunits (Rac1, p47^phox^ and p67^phox^), decreased NOX-dependent superoxide formation and increased the bioavailability of NO (23).

The Nrf2-mediated regulation of cellular antioxidant mechanisms plays an essential role in defense against oxidative stress due to its coordinated induction of genes encoding several antioxidants and detoxifying enzymes such as catalase, superoxide dismutase (SOD) and HO-1 (45). Our study revealed that tenofovir-treated rats showed an increase in the renal expression of Nrf2 and HO-1 probably due to the development of oxidative stress in this group. Administration of nebivolol normalized the expression of these parameters, since this drug reestablished the levels of TBARS and GSH in the TDF+NBV rats compared to C and NBV rats. It is important to highlight that constitutive Nrf2 activity is essential in the maintenance of redox balance under basal condition and its induction occurs in response to oxidative stress. Therefore, with the appearance of cell injury, Nrf2 is translocated to the nucleus and stimulates the production of cytoprotective genes (46–48). According to our findings, nebivolol may mitigate oxidative stress-mediated kidney injury in tenofovir-induced nephrotoxicity by modulating Nfr2/HO1 pathway.

In addition to the participation of the Nrf2/HO-1 signaling pathway, mitochondrial oxidative stress may be straightly associated with mechanisms underlying the arising and progression of both AKI and CKD (49). Mitochondria hold ROS scavenging systems through the conversion of O_2_ into H_2_O_2_ by the actions of SOD, such as Cu/Zn-SOD and MnSOD. We demonstrated that tenofovir-treated rats presented an increase in MnSOD renal expression, probably in response to a higher oxidative stress. Nebivolol treatment restored this enzyme expression in the TDF+NBV rats compared to the C and NBV rats. Tenofovir-induced nephrotoxicity has been related to structural mitochondria abnormalities including wide alterations in size and shape, resulting in disruption on mitochondrial DNA and development of oxidative stress (4, 50). In agreement with our study, Mustafi et al. reported that increased metabolic rates and mitochondrial dysfunction contributed to the elevation of ROS concentration, which was suppressed by the increase of endogenous MnSOD levels in cancerous cells (51).

Altogether, our findings demonstrated that tenofovir-treated rats showed an increase in the antioxidant elements (Nrf2, HO-1 and MnSOD) possibly in response to an imbalance between TBARS and GSH levels, suggesting that oxidative stress activated both mitochondrial enzymatic system and Nrf2/HO-1 signaling pathway. Nevertheless, experimental studies have shown that despite the presence of intense oxidative stress, both Nrf2 and its cytoprotective gene HO-1 activities were downregulated, indicating that the injured kidney paradoxically compromised the Nrf2/HO-1 signaling pathway activation (45, 48, 52). Although Nrf2, HO-1 and MnSOD are considered protective molecules, TDF+NBV rats showed a decrease in the renal expression of these elements. This observation could be explained by both the potent antioxidant action of nebivolol and the moderate kidney injury found in this group, since HO-1 is a heat shock protein responsive to tissue damage (53).

In summary, nebivolol was capable of attenuating tenofovir-induced nephrotoxicity through its involvement in the NO cascade modulation and RAAS activity maintenance. Moreover, nebivolol had an outstanding systemic and renal antioxidant effect, as well as notable anti-inflammatory and anti-fibrotic properties. Hence, our study indicates that administration of nebivolol may be a beneficial therapeutic strategy to slow the progression of renal disease in patients undergoing tenofovir treatment and may offer a better prognosis and an improvement in quality of life of individuals living with HIV/HBV.

## Data availability statement

The original contributions presented in the study are included in the article, further inquiries can be directed to the corresponding author.

## Ethics statement

The animal study was reviewed and approved by Comissão de Ética no Uso de Animais (CEUA) do Hospital das Clínicas da Faculdade de Medicina da Universidade de São Paulo—protocol n. 1287/2019.

## Author contributions

Conceptualization: MN and DC. Data curation: MN, DB, MM, AB, RV, and DC. Investigation and validation: MN, AB, RV, and DC. Writing and reviewing the original drafting: MN, AB, RV, and DC. Editing: AS. All authors approved the submitted version.

## Funding

This work was supported by the Fundação de Amparo à Pesquisa do Estado de São Paulo - FAPESP; grant numbers 2018/04930-6 (ACB), 2018/12297-1 (RAV), 2019/20840-0 (DC).

## Conflict of interest

The authors declare that the research was conducted in the absence of any commercial or financial relationships that could be construed as a potential conflict of interest.

## Publisher's note

All claims expressed in this article are solely those of the authors and do not necessarily represent those of their affiliated organizations, or those of the publisher, the editors and the reviewers. Any product that may be evaluated in this article, or claim that may be made by its manufacturer, is not guaranteed or endorsed by the publisher.
